# On the importance of sensor height variation for detection of magnetic labels by magnetoresistive sensors

**DOI:** 10.1038/srep12282

**Published:** 2015-07-21

**Authors:** Anders Dahl Henriksen, Shan Xiang Wang, Mikkel Fougt Hansen

**Affiliations:** 1 Department of Micro- and Nanotechnology, Technical University of Denmark, DTU Nanotech, Building 345 East, DK-2800 Kongens Lyngby, Denmark; 2 Department of Materials Science and Engineering, Stanford University, California 94305, USA; 3 Department of Electrical Engineering, Stanford University, California 94305, USA

## Abstract

Magnetoresistive sensors are widely used for biosensing by detecting the signal from magnetic labels bound to a functionalized area that usually covers the entire sensor structure. Magnetic labels magnetized by a homogeneous applied magnetic field weaken and strengthen the applied field when they are over and outside the sensor area, respectively, and the detailed origin of the sensor signal in experimental studies has not been clarified. We systematically analyze the signal from both a single sensor stripe and an array of sensor stripes as function of the geometrical parameters of the sensor stripes as well as the distribution of magnetic labels over the stripes. We show that the signal from sensor stripes with a uniform protective coating, contrary to conventional wisdom in the field, is usually dominated by the contribution from magnetic labels *between* the sensor stripes rather than by the labels on top of the sensor stripes because these are at a lower height. We therefore propose a shift of paradigm to maximize the signal due to magnetic labels between sensor stripes. Guidelines for this optimization are provided and illustrated for an experimental case from the literature.

In recent years, much research has gone into performing molecular diagnostics with magnetic sensors and employing magnetic labels as an alternative to fluorescence readout^1^,^2^. Magnetic sensors detect the presence of magnetic labels that are magnetized by either an externally applied field^3^,^4^,^5^,^6^, the magnetic field created by the sensor current^7^,^8^,^9^, or by on-chip current lines^10^. Improvements in cleanroom fabrication procedures have enabled sensors with sizes in the micro- and nano-regime. A sandwich assay is most often used to achieve high specificity and affinity towards the biological target. Here, antibody or DNA capture-probes are spotted on the sensor and the target is labeled with magnetic beads. Often, however, the capture probe immobilization procedure is not limited to the surface of the microsized sensor, and instead probes are uniformly present on both the sensor surface and its surroundings. For fluorescence detection the signal increases with the amount of fluorophores, and a uniform dense layer of fluorescent labels is optimal for detection. However, for magnetic labels the signal depends on the positions of the labels relative to the sensor.

For magnetic beads magnetized by an external magnetic field, the signal from some beads will cancel that from other beads. This cancellation originates from the rotating nature of the magnetic dipole field, i.e., the dipole-field below or adjacent to a bead is anti-parallel to the field in front of the bead. Therefore, the fields from two differently placed beads can partially cancel each other. In the limit of a perfect flat monolayer of beads above the sensor, the fields from the beads will cancel out perfectly, and thus there will be no sensor signal^11^.

The magnetic field from a single homogeneously magnetized bead is known exactly to be the dipole field, but when more than one bead are present the beads may interact and the total magnetic field is not easily calculated. As the magnetic dipole field from each bead drops off as 1/*r*^3^ the following assumptions are usually made in the literature to simplify the theoretical treatment:

(1) Beads outside the sensor surface do not contribute any signal, and beads on top of the sensor can be approximated as a single dipole positioned at the center of the sensor^12^,^13^,^14^,^15^,^16^.

(2) For sensors consisting an array of stripes, the separation between the stripes is so large that a magnetic bead only interacts with one sensor stripe^16^.

(3) Bead-bead interactions can be neglected^13^,^14^,^15^,^16^.

In this paper, we perform a systematic theoretical analysis and show that some of these assumptions are inadequate. We then optimize the geometry of an arbitrary array of sensor stripes considering that magnetic beads can be bound both on top of and between the sensor stripes. We will show that the magnetic beads located *outside* the sensor stripes usually dominate the detected signal, while the beads located on top of the sensor stripes reduce this signal. These results explain both the sign and the magnitude of the sensor signals observed for a uniform coverage of magnetic beads. Moreover, we show that an increase of the height separation between beads on top of and outside the sensor or a decrease of the sensor width, may increase the signal. Such proposed design considerations reduce the signal cancelation of beads on top of and outside the sensor stripes and increase the overall system sensitivity.

## Theory

### Assumptions and definitions

#### Magnetic beads

We will assume the magnetic beads to be superparamagnetic with constant magnetic susceptibility *χ* and no magnetic remanence. Moreover, we assume all beads to be magnetized by a homogeneous external field applied in the *y*-direction creating a magnetic moment *m*_*y*_ in each bead. In this external field, each magnetic bead produces a dipole field that perturbs the external field experienced by the sensor.

#### Magnetic field sensors

**Figure 1:**
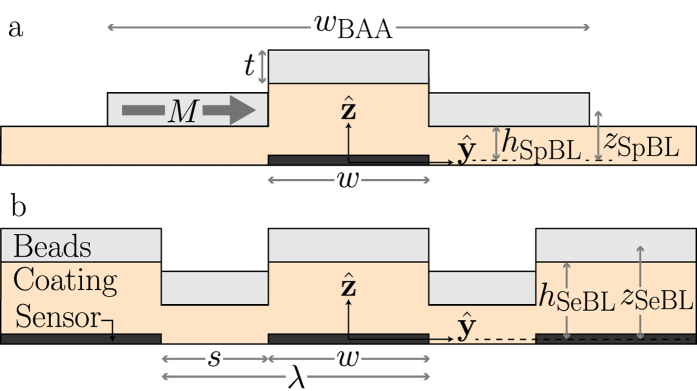
Illustration of cross-section in the *yz*-plane of (**a**) the single sensor stripe geometry, and (**b**) the periodic sensor stripe array geometry with definition of geometrical parameters and the coordinate system.

Magnetoresistive (MR) sensors show great promise as the emerging technology for magnetic biodetection. These sensors usually consist of a magnetic stack in a stripe geometry with a resistance that depends linearly on the experienced field. We will assume that the sensor geometry is either a single stripe^17^ or an array of stripes^6^ aligned along the *x*-direction. Often, the stripe length is much larger than the stripe width and we will therefore assume that the stripes are infinitely long such that the problem is reduced to two dimensions in the *yz*-plane (Fig. 1). Moreover, we will assume that the sensor detects the average value of the *y*-component *H*_*y*_ of the total magnetic field experienced by the sensor. Usually, the presence of magnetic beads attached to the biologically active area (BAA) is detected by comparing the average magnetic field detected by the sample sensor with that detected by a reference sensor such that the resulting sensor signal *S* can be written as


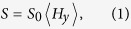


where *S*_0_ is a proportionality factor and 〈*H*_*y*_〉 is the average magnetic field experienced by the sensor due to the magnetic beads.

#### Magnetic plate approximation and geometrical parameters

Magnetic beads will generally be present both on top of and beside the magnetic sensor stripes on the biologically active area, BAA. Assuming that beads bind randomly to the BAA, the expected value of the sensor signal due to beads bound on the BAA can be obtained by integrating the signal over all possible bead positions in the BAA while taking into account the bead packing density. An alternative approach is to abandon the point dipole approximation for a single bead and approximate the bead distribution with a plate with a thickness *t* identical to the magnetic bead diameter. The magnetization of these plates depends on the number of beads per area and the magnetic bead magnetization. The plates will be assumed to have a constant magnetization, **M** = ***M*****ŷ** and to have infinite length in the *x*-direction, corresponding to the assumption that the stripes are much longer than they are wide.

Figure 1 shows an illustration of the *yz*-plane cross-section of both a single sensor stripe and a part of a sensor array with infinitely many stripes. In both cases, we define the origin of the coordinate system to be in the middle of the active layer of the sensor stack. We assume this layer to be so thin that the variation of the magnetic field over the thickness of the layer can be neglected. The plate from beads on the BAA is assumed to be placed symmetrically with respect to *y* = 0 and it has a width *w*_BAA_. Over the sensor stripes, the plates are placed with their vertical centers at *z*_SeBL_ corresponding to spacers over the sensors of thickness *h*_SeBL_ (*z*_SeBL_ = *h*_SeBL_ + *t*/2), where SeBL denotes ‘sensor bead layer’. Correspondingly, the plates outside the sensor stripes are placed with their vertical centers at *z*_SpBL_ corresponding to spacers outside the sensors of thickness *h*_SpBL_ (*z*_SpBL_ = *h*_SpBL_ + *t*/2), where SpBL denotes ‘spacer bead layer’.

A single sensor stripe (Fig. 1a) has the width *w*. For arrays (Fig. 1b) all sensor stripes have width *w* and period *λ* = *w* + *s*, where *s* is the edge-to-edge separation of two stripes. The response from a sensor centered at *y* = 0 in this 2D description is generally given by


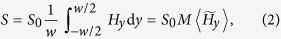


with 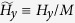. Here, we used that the magnetic field is proportional to the magnetization *M*, thus the sensor designs aim to maximize the average normalized field 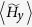.

### Magnetic field calculation

#### Single bead

A single homogeneously magnetized magnetic bead with its center at **r**_0_ produces a dipole magnetic field at **r** given by


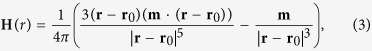


where **m** is the magnetic moment of the bead.

#### Magnetic plate(s)

The magnetic field from a single homogeneously magnetized plate with a magnetization **M** can be calculated using the Biot-Savart law, where the plate magnetization is represented as bound surface currents **J** = ∇ × **M**. In the Supplementary Information, Section S1, we use this approach to derive an analytical expression for the magnetic field from a single plate, which can easily be extended by superposition to obtain the magnetic field from any finite array of SeBLs and SpBLs.

For a periodic array of magnetic plates, the integration approach gives rise to an infinite series of current contributions. To avoid this, the magnetic potential can be considered and the Ampère-Maxwell differential equation can be solved for the magnetic field within a box with periodic boundary conditions. In the Supplementary Information, Section S1, we derive an analytical expression for the magnetic field from a periodic array of homogeneously magnetized plates. This expression can be used to obtain the magnetic field for any periodic array of magnetized plates by superposition of the SeBL and SpBL contributions to the magnetic field.

#### General properties of the magnetic field from an infinite array of magnetized plates

The *x*-component of the magnetic field from a plate geometry, which is infinite in the *x*-direction is zero, whereas the *y*- and *z*-components of the magnetic field resemble those from a bar magnet with field lines going from one pole (the face of the magnetic plate) to the opposite pole either of the plate itself or an adjacent plate. While the equations in Supplementary Info Section S1 describing the magnetic field are complex, some important attributes of the field are easily obtained. Throughout this paper the following will be used:

(1) The field generated by the SeBL is minus the field generated by the SpBL if the layers are at the same height (*z*_SeBL_ = *z*_SpBL_):


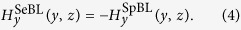


Here, 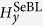 and 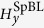 are the magnetic fields from only the SeBL and SpBL, respectively. This is true for all geometries and is easily proven by superposing a plate with magnetization −*M*. Therefore, an infinite plate with no height difference between the SpBL and SeBL must give zero magnetic field.

(2) The *y*-integral of *H*_*y*_ over a period for a fixed value of *z* is zero, ∫_*λ*_*H*_*y*_d*y* = 0. This implies that


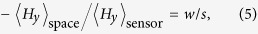


where 〈*H*_*y*_〉_sensor_ and 〈*H*_*y*_〉_space_ denote the average magnetic fields acting on the sensor stripe and on the space between the sensor stripes, respectively. Thus, changing *w*/*s* can be used to squeeze more or less field into the sensor region. As Eq. (5) holds for, e.g., the SpBL and because *w*/*s* is positive, the SpBL and SeBL always generate signals of opposite signs.

(3) Outside a magnetized plate, the magnetic field is subject to the Laplace equation, ∇^2^*H*_*y*_ = 0 with periodic boundary conditions. The solution *H*_*y*_ can be written as a sum of products of periodic functions in the *y*-direction and exponentially decreasing functions in the *z*-direction:


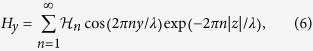


where H_n_ are constants. The slowest decreasing function is the fundamental mode (*n* = 1) and thus as *z* increases 〈*H*_*y*_〉 becomes proportional to exp(−2*π*|*z*|/*λ*).

Finally, we also note that the magnetic field **H**(*y*, *z*) is unchanged if all geometrical parameters, including the point of observation (*y*, *z*), maintain a constant ratio to the period, i.e., the field dependence can be reduced to 
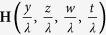
. Therefore, all geometrical parameters can be normalized with the period *λ* while keeping the results general. The normalized parameters will be denoted as 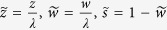, and 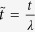.

## Results and Discussion

### Single sensor stripe

We first discuss the signal dependence on the position of a single magnetic bead for a single sensor stripe. Next, we discuss the signal from a single sensor stripe covered by a bead layer of finite size and the effect of the size of the bead layer as well as a height difference between the bead layer over and outside the sensor area.

**Figure 2:**
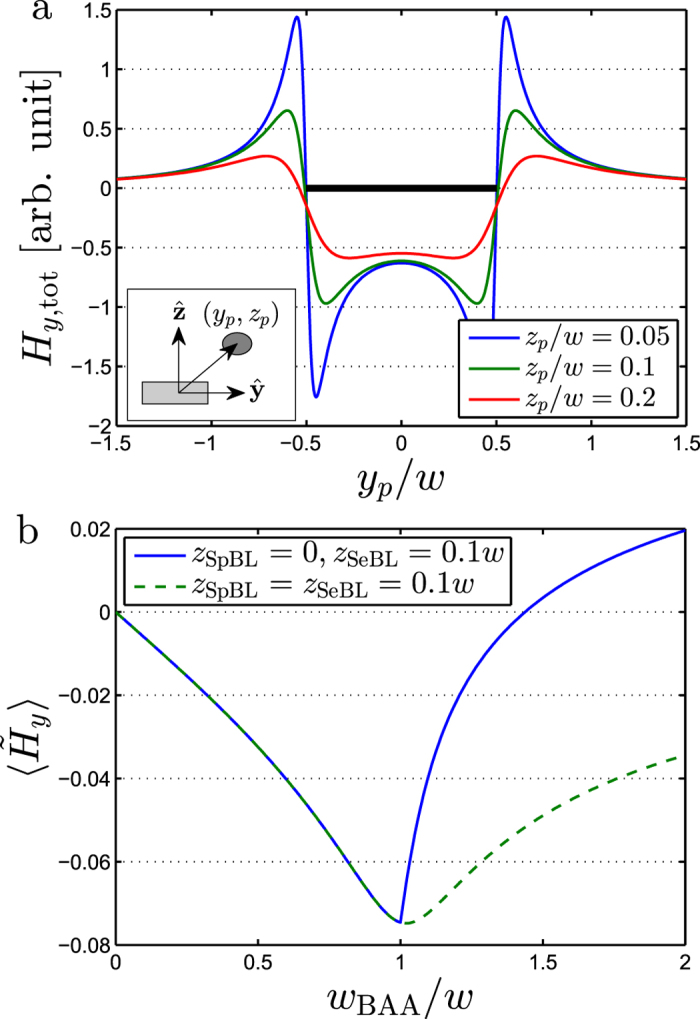
(**a**) The normalized *y*-component of the total magnetic field from a single magnetic bead for an infinitely long sensor of width *w* as function of the position of the bead. The bead is magnetized along the *y*-direction with magnetic moment *m*_*y*_ and is positioned at (*y*_*p*_, *z*_*p*_) with respect to the sensor centroid. (**b**) The mean field 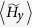 acting on a single sensor stripe as a function of the normalized width *w*_BAA_ of the biologically active area. The calculation was done for *t*/*w* = 0.1 and the indicated values of *z*_SeBL_ and *z*_SpBL_.

The MR sensor detects the presence of magnetic beads via their perturbation of the externally applied magnetic field. However, both the magnitude and sign of the signal due to a single magnetic bead depend on the position of the bead relative to the sensor stripe. This is illustrated in Fig. 2a, where the signal from a single bead is plotted as function of its position. As can be seen in Fig. 2a, the bead may provide a positive or negative field depending on whether it is positioned on top of or outside the sensor stripe. Moreover, beads with lower values of 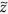 provide a stronger field.

The magnetic bead signal can be optimized by limiting the BAA to cover only the sensor stripe. However, often this is not practically possible and beads will be present in some area around the sensor stripe. This is modeled in Fig. 2b, where the bead signal is plotted as a function of the relative width of the biological active area *w*_BAA_/*w*. When the beads on top of the sensor area are at the same height as beads outside the sensor area (Fig. 2b, dashed line), the signal amplitude decreases when *w*_BAA_/*w* increases beyond 1 and 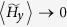 for *w*_BAA_/*w* → ∞. However, if the beads outside the sensor area are at a lower height (*z*_SpBL_ < *z*_SeBL_), the sensor signal changes sign and remains non-zero as *w*_BAA_/*w* increases (Fig. 2b, solid line).

Figure 2 clearly illustrates that it is generally insufficient to only consider the beads on top of the sensor, unless the biologically active area can be strictly limited to the sensor surface. When beads are present outside the sensor stripe, they influence the magnitude and possibly also the sign of the expected signal.

### Finite array of sensor stripes and magnetic bead layers

**Figure 3:**
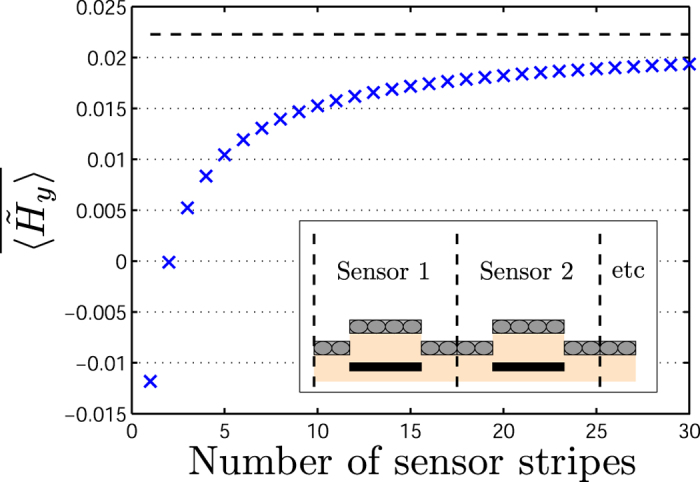
The average signal from a variable number *N* of sensor stripes when magnetic beads are present for −*λ*/2 ≤ *y* − *y*_*k*_ ≤ *λ/*2 for sensors with center positions *y*_*k*_. The dashed line shows the result for *N* = ∞. The calculation was done for 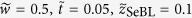 and 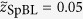.

We consider a finite array of sensor stripes to elucidate how the signal depends on the number of sensor stripes for a finite size of the bead layer. To limit the parameter space, we assume the bead layer to cover a full period *λ* centered over each sensor stripe (i.e., between the vertical dashed lines in the inset of Fig. 3).

For a magnetic bead layer at a constant height (*z*_SeBL_ = *z*_SpBL_) an infinite number of beads outside the sensor area is required to cancel the signal contribution from the beads on the sensor area, (cf. Fig. 2b for *w*_BAA_ → ∞). However, for a sensor array with a height difference between the sensor and spacer bead layers (*z*_SpBL_ ≠ *z*_SeBL_), the lower layer usually dominates the sensor signal. This is illustrated in Fig. 3 that shows the signal 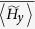 averaged over all sensor stripes as function of the number *N* of sensor stripes for the indicated values of 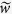, 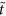 and 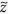.

For increasing *N*, the amount of beads outside each sensor area increases and reduces the signal from the beads over the area of the sensor. For the example in Fig. 3, the average signal changes sign from negative to positive when *N* ≈ 2, so even for such small arrays the signal from the SpBL dominates that from the SeBL. Further, when *N* > 30, the averaged field resembles that for *N* = ∞. In the remainder of this paper, we will focus on the results for *N* = ∞.

### Infinite array of sensor stripes

**Figure 4:**
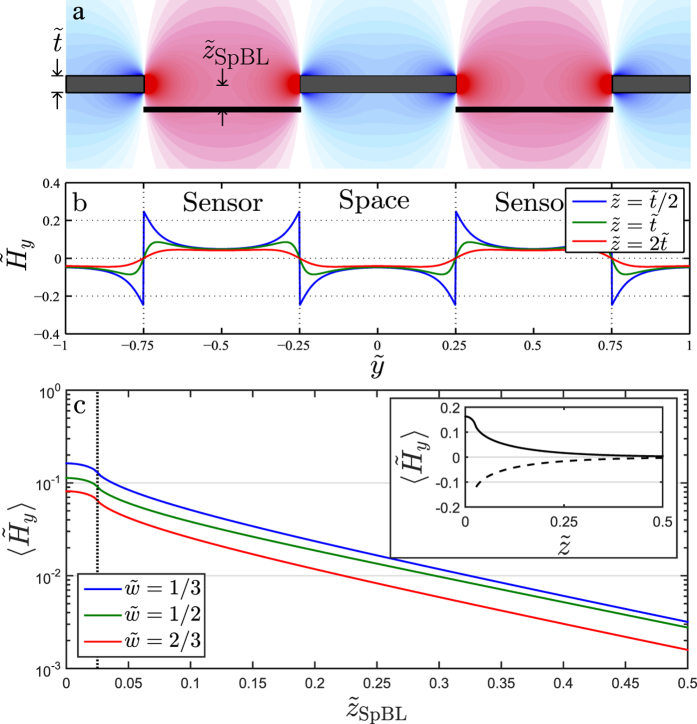
Calculations of normalized magnetic field 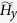 from an infinite SpBL array. (**a**) Color map of 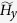. The sensor stripes placed at 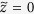 are indicated by the black bars. Red and blue colors indicate positive and negative field values, respectively. (**b**) Values of 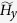 at 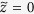 vs. 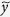 for the indicated values of 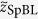. (**c**) Average normalized magnetic field 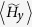 vs. 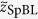 for the indicated values of 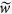. The inset shows 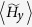 on a linear scale vs. 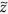 for SpBL (solid line) and SeBL (dashed line) arrays, respectively, for a geometry with 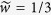. All calculations were done with 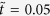.

We consider an infinite array of sensor stripes and first discuss general properties of the magnetic field. Figure 4a shows an example of 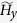 calculated for an array of SpBL with no SeBL. The field is strongest near the poles (faces) of the bead layer and it displays a high degree of symmetry. An SpBL/SeBL array provides a positive/negative 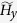 in the sensor stripe, respectively. When considering the field from any layer, the shape of 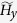 across the sensor depends on the heights 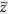 of the bead layers as illustrated in Fig. 4b. For low 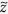-values the SpBL gives rise to a strong field at the edge of the sensor. However, when 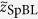 increases and becomes larger than 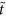 and comparable to 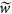, the fields from different poles will overlap. This results in a reduction of the overall field strength, but the resulting 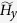 will be more uniform and assumes its maximum value over the middle of the sensor stripe.

Below, we study the dependence of the sensor signal vs. 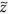 for either an SpBL or an SeBL, and when the two contributions are combined. Finally, we study the influence of 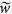 and discuss how the sensor geometry can be chosen to maximize the sensor response to a complete magnetic bead layer.

#### Effect of sensor distance to bead layer, 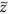 . 

While the shape of 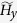 has a complicated dependence on 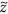, the average field 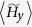 is a simple function of 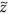. Generally, 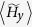 decreases with 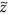. This is illustrated in Fig. 4c, where 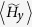 is evaluated as a function of 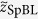 for an SpBL array with 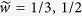 and 2/3. Note, that the red curve for 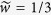 assumes half the value of the blue curve obtained for 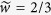 in agreement with the prediction of Eq. (5). When 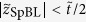, i.e., when the bead layer is in line with the sensor layer, the field decreases slowly with increasing 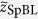. In the Supplementary Information Section S2 it is shown that the detailed plate approximation is only important in this limit. When 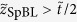, the value of 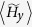 first decreases rapidly followed by an exponential decrease towards zero, which appears as a straight line in the semi-logarithmic plot in Fig. 4c.

As previously discussed, the SeBL generates the same field as the SpBL but with opposite sign. This is illustrated in the inset of Fig. 4c, where the average fields from the SpBL (solid line) and the SeBL (dashed line) are plotted as function of 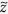. Note, that for 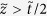 the only difference is the sign of the average field. As the fields from the SpBL and SeBL have opposite signs, they partially cancel each other for all geometries with both a SeBL and a SpBL. To maximize the sensor signal, one should therefore increase the contribution from one layer while decreasing the contribution from the other. As both fields diminish with the height over the sensor layer, the signal is maximized by having one layer as close as possible to the sensor and one layer as distant as design permits.

Practically, however, it may be difficult to obtain a large difference between 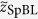 and 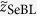. Let us write 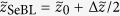 and 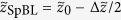. For 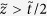 using the first order (*n* = 1) term of Eq. (6), we can approximate the magnitude of the field on the sensor from the SpBL to


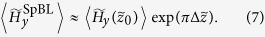


The contribution from the SeBL has the opposite sign of 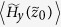 and the opposite sign of 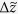. Combining the two contributions, we obtain


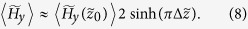


**Figure 5:**
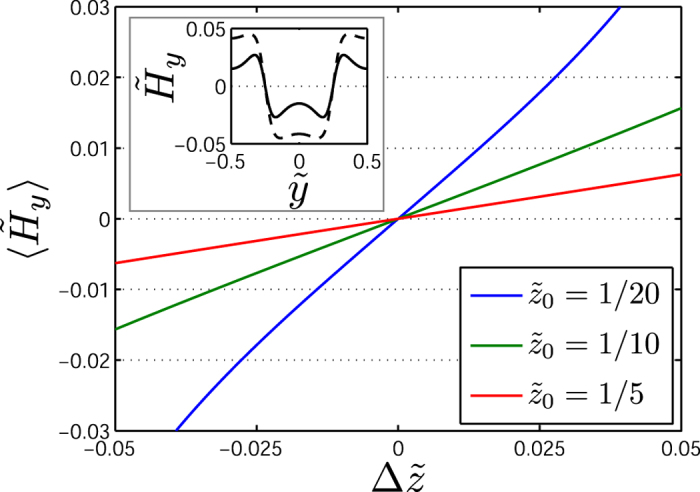
The average field on the sensor as a function of the separation distance of the SeBL and SpBL. The inset shows the magnetic field from only the SpBL (dashed line) and from both layers (solid line). All calculations were done for 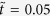 and 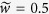, and fo r the inset 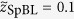 and 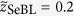.

When the SeBL and the SpBL are present at approximately the same height 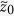 over the sensor, the sensor signal will depend linearly on 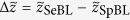 with a slope that increases for decreasing 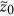. This is shown in Fig. 5 where values of 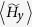 are calculated for the combined SpBL and SeBL as function of 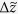 for the indicated values of 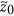. The inset of Fig. 5 shows an example of the profile of 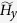 calculated for only the SpBL (dashed line) and for both layers (solid line), where the general reduction of 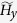 when both layers are present is clearly observed.

#### Optimization of sensor width, 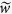 . 

**Figure 6:**
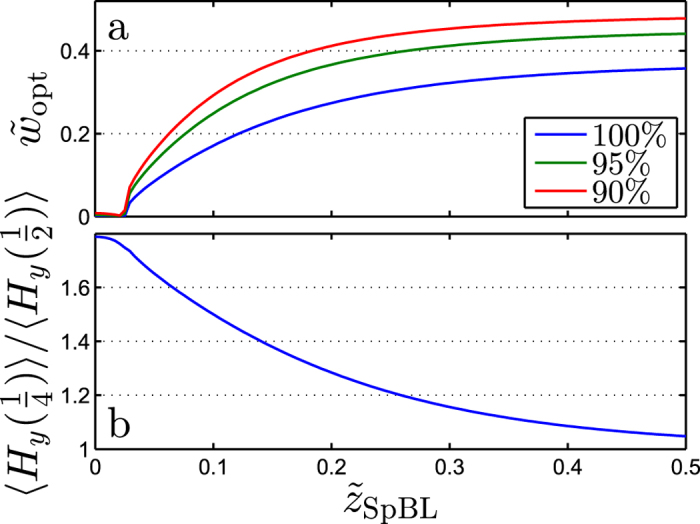
(**a**) The optimal normalized sensor width 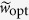 found as function of the bead layer height 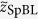 for an infinite SpBL array. The values obtained for 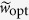 correspond to 100%. The figure also show the values of *w* corresponding to 90% and 95%. (**b**) The gain from using a reduced width, 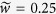, instead of 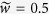 for an infinite SpBL array. All calculations were done for 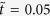.

Of the sensor widths investigated in Fig. 4c, 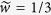 provided the strongest field in the sensor area. When 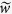 is reduced, the distance between the magnetic material of adjacent SpBLs decreases and the strength of the magnetic field over a sensor stripe increases. This is analogous to the increase of the magnetic field in an air gap between two magnetic plates when their separation is reduced. However, a smaller gap between the magnets also makes the field more localized near the gap such that the field away from the gap is reduced. Therefore, the width 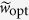 that produces the highest signal for a given SpBL depends on both 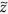 and 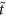 such that a small value of 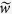 is preferred in the near-field limit when 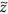 is small and a comparatively larger value of 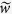 is preferred in the far-field limit when 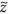 is large. This is illustrated in Fig. 6a, where 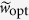 is found by numerical optimization for an infinite SpBL array as function of 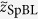 for 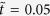. When the bead layer is close to the sensor layer (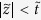), the sensor output can be considerably enhanced by reducing 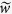. For example, if 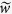 is reduced from 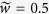 to 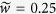 for 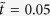, the average magnetic field acting on a sensor stripe is increased by 80% for 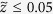 (Fig. 6b). Further, when the sensor is in line with the SpBL, 〈*H*_*y*_〉 increases towards *M* as *w* decreases towards zero. Figure 6a also shows the curves of 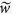 that give 90% and 95% of the signal obtained for 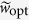. These illustrate the impact of changing 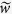 towards 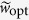. When both SeBL and SpBL arrays are present, changing 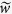 towards 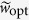 increases the field from the layer with the lowest value of 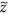 more than that from the other layer, thus still increasing the overall sensor response.

#### Signal optimization

We have found that the sensor signal can be increased by: (1) Reducing the average height 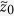 of the SeBL and SpBL; (2) increasing the separation 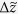 between the SeBL and SpBL; (3) decreasing the sensor width 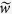 towards 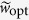 (cf. Fig. 6); (4) using magnetic beads with a higher magnetization (signal is proportional to *M*) or a larger size (signal increases with 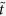).

Decreasing 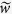 will be most beneficial for the lowest layer. Likewise, in all practical systems we have 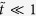 and increasing 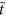 will also be most beneficial for the lowest layer; both adjustments will thus also increase the signal from a combined SeBL and SpBL.

A more radical approach to signal maximization is to reduce the signal due to beads on top of the sensor area, for example by coating the sensor surface differently to prevent or reduce bead binding. This approach presents a shift of paradigm and differs substantially from common practice in the literature where focus has been on binding beads on top of the sensor area. Moreover, for spin valve sensors, for example, the free magnetic layer will have a magnetization component in the *y*-direction, and its stray field, in practice, affects the moment of the magnetic beads^2^. This sensor stray field will reduce the moment of the SeBL and enhance the moment of the SpBL. This effect, depending on the exact magnetic excitation, may also enhance the signal from the SpBL compared to that from the SeBL. A potential disadvantage of using a scheme based on only a SpBL is that the magnetic beads may be less accessible to a liquid flow and become trapped near steps in the surface height.

### Practical sensor design considerations

Next, we consider consequences for practical sensor designs. We consider an infinite array of sensor stripes and assume that the sensor stripe width *w* is fixed by the sensor design. For a spin valve sensor, for example, the shape anisotropy may be used to control the magnetization of the free layer and that constrains the sensor width. We moreover assume that the minimum thickness of the protective sensor coating is fixed to *h*_SeBL_ by operation requirements of the sensor and that the bead size *t* is given and fixed. The parameters under our control are the sensor spacing *s* and the heights *z*_SeBL_ and *z*_SpBL_, where *z*_SeBL_ ≥ *h*_SeBL_ + *t*/2.

We first note that an increase of *s* results in a decrease of 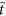, 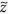 and 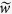. A decrease of 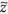 and 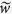 will generally increase the signal, whereas a decrease of 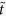 will generally decrease the signal. Thus, the combined effect of changing *s* is not clear from the previous analysis in terms of the normalized variables, where the normalization with the period was chosen to maintain the results in a general form.

**Figure 7:**
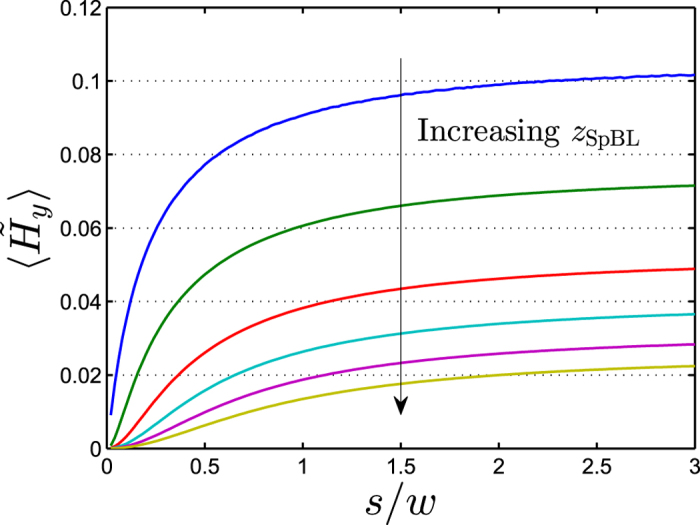
The average magnetic field 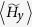 on the sensor for an infinite SpBL array calculated as function of *s*/*w* for *z*_SpBL_/*w* = 0.05, 0.1, 0.2, 0.3, 0.4 and 0.5. Calculations were done for *t*/*w* = 0.1.

For the above situation where *w* and *t* are fixed and *s* and *z* can be varied, it is more convenient to discuss results normalized to *w*. Figure 7 shows values of 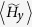 calculated for an infinite SpBL array as function of *s*/*w* for the indicated values of *z*_SpBL_/*w*. Figure 7 can be used to understand how *s* and *z*_SpBL_ modify the signal. For a fixed value of *z*_SpBL_, we observe a steep increase of 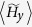 with *s* that flattens out when *s*/*w* ~ 1 and 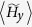 becomes essentially independent of *s* for *s*/*w* > 2. In this limit, the majority of the magnetic field lines, from the SpBL North face, cross the sensor space to terminate on the South face of the next SpBL and thus only little can be gained by increasing *s*. Choosing a fixed value of *s*/*w* (e.g. *s*/*w* = 2 and changing *z*_SpBL_/*w*), we observe that the signal increases strongly for small values of *z*_SpBL_ > *t*/2 in agreement with the exponential dependence on *z*_SpBL_ in Fig. 4. As 
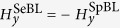
 for *z* > *t*/2 the effect of the combined SpBL and SeBL arrays is obtained by subtracting curves for their corresponding *z*-values. As can be seen in Fig. 7, the difference between curves at different heights is mostly unchanged for *s* ≥ *w*; thus when both the SpBL and SeBL arrays are present, *s* ≥ *w* is close to optimal and a smaller spacing will decrease sensor signal. Thus only increasing *s* beyond *w* is not a feasible approach for reducing 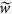 towards 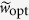.

### Case study

In this section, we analyze the signal for a concrete experimental case and discuss the potential to increase the sensor signal by changing sensor design parameters and sensor functionalization.

We will discuss the spin-valve (SV) geometry, which has been used by Gaster *et al.* to detect proteins and other biomarkers in attomolar to femtomolar concentrations^6^. Their sensor design is based on an array of *N* = 32, SV resistor stripes of a length of 100 m and a width of *w* = 0.75 m detecting the *y*-component of the magnetic field due to magnetic beads. This sensor array is large enough to justify the use of the results obtained for an infinite array of sensor stripes. The BAA of the sensor was significantly larger than the sensor area and magnetic beads were present over the entire BAA with a nominally uniform density. The geometrical parameters of the sensor geometry relevant for the calculations are given in Table 1. Further details on the sensor design and geometry are given in the Supplementary Information Section S3.

We first analyze the sensor signal and assume that the sensor array and the BAA are infinitely large. For the parameters in Table 1, we calculate 
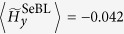
 and 
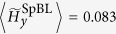
 corresponding to 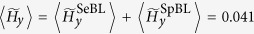. Thus, the analysis predicts that the sensor signal is dominated by the SpBL contribution and the presence of magnetic beads strengthens the sensor response to an externally applied magnetic field. The experimental studies by Gaster *et al.*^6^ used an electronic readout scheme that gives the signal magnitude and not the signal sign and hence those studies cannot be used to verify the presented theoretical results. In the Supplementary Information, Section S4, we present measurements of the response of a sensor vs. applied magnetic field before and after exposure to a concentrated magnetic bead suspension. The results, although preliminary, clearly show that the presence of magnetic beads strengthens the response of the sensor to the applied magnetic field and hence that magnetic beads located outside the sensor stripes dominate the sensor signal. Therefore, the results favor the proposed shift of sensing paradigm.

It is noteworthy that the above theoretical result obtained for the combined SpBL and SeBL array has approximately the same magnitude (but opposite sign) of that due to solely the SeBL array, whereas the result for solely the SpBL array is about twice that obtained for the combined SpBL and SeBL array. Thus, the theoretical analysis predicts that no signal improvement may be obtained for the present design by limiting the beads to be present only on top of the sensor stripes whereas a factor of two signal improvement can be obtained by limiting the beads to be present only outside the sensor stripes. This could potentially be achieved by a selective functionalization of the chip surface outside the sensor stripes. Another approach is to further increase the height difference between the sensor stripe and the space between the stripes either by adding extra coating on the sensor stripe or by removing material in the spacer region to allow the magnetic bead layer to be at approximately the same height as the active layer of the sensor. Calculating the signals for *z*_SpBL_ = 0 and an unchanged value of *z*_SeBL_, we obtain 
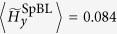
, i.e., the improvement compared to the current value of *z*_SpBL_ is 2% and thus only little can be gained by decreasing *z*_SpBL_. The recommendation of increasing *z*_SeBL_ may result in a significant signal improvement, but due to the risk of unspecific trapping of beads it will have to stand the test of experiments to prove its practicality.

We also note that the width and space of the sensor stripes correspond to 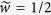 (*w* = *s*). Comparing to Fig. 7, it is observed that this value is close to optimal and only little improvement of the signal is expected upon a further increase of *s*. Indeed, the effect of increasing *s* from *s* = *w* to *s* = 2*w* results in 
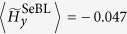
 and 
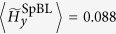
 corresponding to changes of 13% and 7%, respectively, and 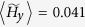, i.e., there is virtually no change of the resulting field from the combined SeBL and SpBL array.

Thus, we conclude that the present sensor design is close to optimal and that improvements of the signal should be sought via selective surface functionalization of the space between the sensor stripes, where a doubling of the signal may be obtained. A detailed experimental verification of the model predictions requires knowledge and control of the magnetic bead distribution as well as a systematic variation of the heights of the magnetic bead layers inside and outside the sensor stripes and is outside the scope of the present work.

## Conclusion

We have analyzed the response of stripe magnetic field sensors to uniform layers of magnetic beads present over the sensor stripes but also outside the sensor stripes when the magnetic beads are magnetized using a homogeneous magnetic field. We have analyzed the signal for a single sensor stripe and for finite and infinite arrays of sensor stripes and have found that the signal produced by the sensor stripes is typically dominated by the magnetic beads *between* the sensor stripes rather than by the beads on top of the sensor stripes as the beads between the sensor stripes are usually at a lower height over the active sensor layer. This provides the first analysis and explanation of the signal origin, sign and magnitude in such sensors.

We have systematically analyzed the influence of geometrical parameters of the sensor stripe array on the sensor output and we have discussed approaches to increase the sensor signal. Based on the analysis, we propose a shift of paradigm to bind magnetic beads to the space between the sensor stripes rather than on top of the sensor stripes as traditionally pursued in the literature as this will enhance the magnetic signal. We have analyzed the sensor signal for a significant case from the literature and validated the signal sign and hence the signal mechanism experimentally. Finally, we have evaluated the effect of implementing the generally suggested approaches to signal improvements. We conclude that the current sensor geometry is close to optimal and predict that a signal improvement by a factor of two may be achieved with a successful implementation of a selective surface functionalization of the space between the sensor stripes. The detailed experimental implementation and verification of these predictions are subjects for future work.

## Additional Information

**How to cite this article**: Henriksen, A. D. *et al.* On the importance of sensor height variation for detection of magnetic labels by magnetoresistive sensors. *Sci. Rep.* **5**, 12282; doi: 10.1038/srep12282 (2015).

## Supplementary Material

Supplementary Information

## Figures and Tables

**Table 1:** Geometrical parameters of spin valve sensors used by Gaster *et al.*^6^.

***w***	***s***	***t***	***z***_**SeBL**_	***z***_**SpBL**_
0.75 μm	0.75 μm	50 nm	69 nm	9 nm
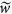	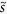	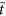	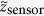	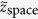
0.5	0.5	0.033	0.046	0.006

See Supplementary Section S3 for more details.
