# Supplementary material for: On the importance of sensor height variation for detection of magnetic labels by magnetoresistive sensors
Source: Sci Rep. 2015 Jul 21;5:12282. doi: 10.1038/srep12282 (PMC4508666; doi:10.1038/srep12282)
Supplement: Supplementary Information [file srep12282-s1.pdf]

Supplementary Information

On the importance of sensor height variation for magnetic bead detection by magnetoresistive sensors

Anders Dahl Henriksen[1], **Shan Xiang Wang**[2,3], **and Mikkel Fougt Hansen**[1,*]

[1]Department of Micro- and Nanotechnology, Technical University of Denmark, DTU Nanotech, Building 345 East, DK-2800 Kongens Lyngby, Denmark
[2]Department of Materials Science and Engineering, Stanford University, California 94305, USA
[3]Department of Electrical Engineering, Stanford University, California 94305, USA
[*]Mikkel.Hansen@nanotech.dtu.dk

S1 Magnetic field calculation

In this section, we present analytical expressions for the magnetic field from a single homogeneously magnetized plate and from a periodic array of a single homogeneously magnetized plate. In both cases, the plates are assumed to be infinitely long in the x-direction and to have a magnetization $\mathbf{M} = M\hat{\mathbf{y}}$

Single plate

The magnetic field at $\mathbf{r} = (x,y,z)$ from a homogeneously magnetized plate can be found by converting the magnetization to a bound surface current $\mathbf{J} = \nabla \times \mathbf{M}$, and calculating the field using the Biot-Savart law

$$\mathbf{H}(\mathbf{r}) = \frac{1}{4\pi}\int_{V_0} \frac{\mathbf{J}\times(\mathbf{r}-\mathbf{r}_0)}{|\mathbf{r}-\mathbf{r}_0|^3}\,\mathrm{d}\mathbf{r}_0 \tag{S1}$$

where V_0 is the volume of the plate. For an infinitely long plate of width W and thickness t centered around $(y,z) = (y_0,z_0)$, which is magnetized in the y-direction, the surface current density is

$$\mathbf{J} = M\big(\delta(z'-\tfrac{t}{2})\left[\theta(y'+\tfrac{W}{2})-\theta(y'-\tfrac{W}{2})\right]-\delta(z'+\tfrac{t}{2})\left[\theta(y'+\tfrac{W}{2})-\theta(y'-\tfrac{W}{2})\right]\big)\hat{\mathbf{x}} \tag{S2}$$

with $(y',z') \equiv (y-y_0, z-z_0)$ and where $\delta(x)$ and $\theta(x)$ denote the Dirac delta and Heaviside step functions, respectively. Combined with Eq. (S1), we obtain

$$\tilde{H}_y = \frac{1}{2\pi}\left[\arctan\left(\frac{y'+\frac{W}{2}}{z'+\frac{t}{2}}\right)-\arctan\left(\frac{y'-\frac{W}{2}}{z'+\frac{t}{2}}\right)-\arctan\left(\frac{y'+\frac{W}{2}}{z'-\frac{t}{2}}\right)+\arctan\left(\frac{y'-\frac{W}{2}}{z'-\frac{t}{2}}\right)\right], \tag{S3}$$

where we recall that $\tilde{H}_y \equiv H_y/M$. Averaging $\tilde{H}_y$ over a sensor stripe of width w with its center at $(y,z) = (0,0)$, we obtain

$$\langle\tilde{H}_y\rangle = \frac{f\left(z_0-\frac{t}{2}\right)-f\left(z_0+\frac{t}{2}\right)}{2\pi w} \tag{S4}$$

with

$$f(z) = g(\tfrac{y_{++}}{z}) - g(\tfrac{y_{+-}}{z}) - g(\tfrac{y_{-+}}{z}) + g(\tfrac{y_{--}}{z}) \tag{S5}$$

$$g(y) = \tfrac{1}{2}\ln\left(1+y^2\right) - y\arctan(y) \tag{S6}$$

$$y_{ij} = y_0 + i\cdot\tfrac{W}{2} + j\cdot\tfrac{w}{2}\ ,\ i\wedge j \in \{-1,+1\}. \tag{S7}$$

This expression can by superposition be used to calculate the field in a single sensor from an arbitrary array of homogeneously magnetized plates.

Periodic plate geometry

The magnetic field from an arbitrary periodic plate geometry can be derived using the following approach: First, the magnetized domain is converted to surface currents; then the field contributions from the different surface currents are split to focus on one of them. The field from one surface current is calculated by using a conformal mapping to the upper half plane, where a simple solution exists. Finally, this is mapped back to the correct geometry.

For periodic geometries, Maxwell's equations are best used in differential form, and the magnetic field can be found by solving the Ampère-Maxwell law for the magnetic vector potential ($\mathbf{A}$). For magnetostatics ($\frac{\partial A}{\partial t} = 0$) the Ampère-Maxwell law simplifies to the three Poisson equations

$$\nabla^2\mathbf{A} = -\mu_0\mathbf{J} \tag{S8}$$

with $\mathbf{J}$ given by Eq. (S2). The geometry can be seen in Fig. S1a, and is formed by rectangular prisms of homogeneously magnetized material of infinite length and arranged in a periodic array. In Fig. S1, the coordinate system, geometrical parameters and magnetization are also defined. If more than one array is present, the magnetic field can be obtained by superposition. As the geometry is periodic we only need to consider a single slab and its magnetization can be converted to two surface currents of magnitude $K = M$ (cf. Fig. S1b).

Each surface current in Fig. S1b can be handled individually and be combined by superposition to obtain the magnetic field from the plate. Translating the coordinate system to the center of the chosen surface current gives a symmetrical geometry and only the domain above the current needs to be considered. In this domain the Poisson equation simplifies to the Laplace equation with known non-zero Neumann boundary condition. By conformal mapping, using the sine function, to the upper-half

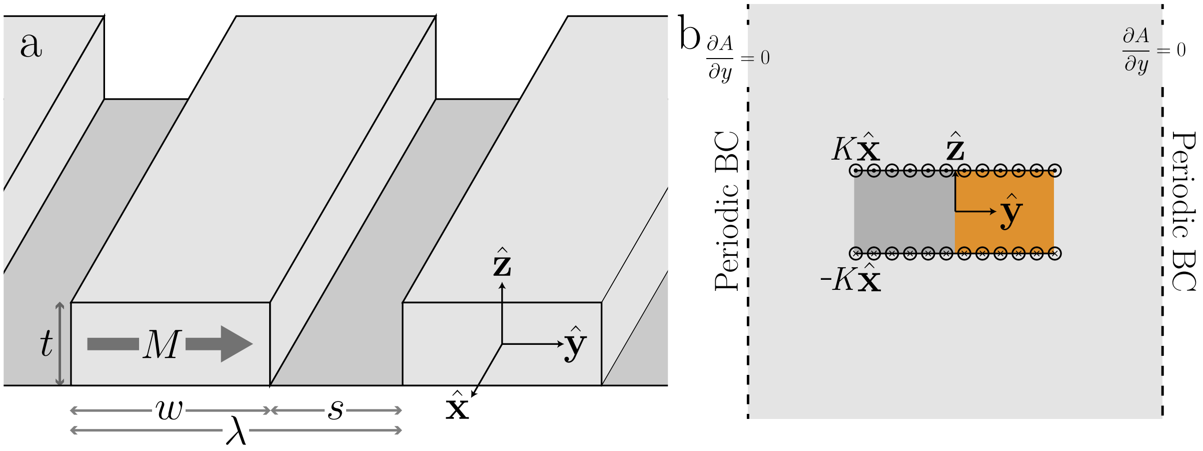

Figure S1. (a) The model used for the theoretical derivation of the stripe field. (b) Illustration of the periodic model with surface currents, **K**, instead of the magnetic domain.

plane, the Laplace equation can be solved using Neumann functions.[1] The solution in the upper-half plane with coordinate (Y,Z) is

$$A_x = \frac{M\mu_0\lambda}{4\pi^2}\int_{-\sin(\frac{\pi w}{2\lambda})}^{\sin(\frac{\pi w}{2\lambda})} \frac{\ln\left((Y-Y_0)^2+Z^2\right)}{\sqrt{1-Y_0^2}}\,\mathrm{d}Y_0 \tag{S9}$$

with

$$Y = \Re\left(\sin\left(\frac{\pi}{\lambda}(y+\mathrm{i}z)\right)\right) \tag{S10}$$

$$Z = \Im\left(\sin\left(\frac{\pi}{\lambda}(y+\mathrm{i}z)\right)\right) \tag{S11}$$

where $\Re$ and $\Im$ denote the real and imaginary parts.

While the integral in Eq. (S9) cannot be evaluated analytically, its derivative, corresponding to the physical magnetic field, can. The resulting magnetic field from a periodic array of slabs with coordinates as in Fig. S1b is given by

$$\mathbf{B}(y,z) = \left(B_{ss,y}(y,z-\tfrac{t}{2}) - B_{ss,y}(y,z+\tfrac{t}{2})\right)\hat{\mathbf{y}} + \left(B_{ss,z}(y,z-\tfrac{t}{2}) - B_{ss,z}(y,z+\tfrac{t}{2})\right)\hat{\mathbf{z}} \tag{S12}$$

with

$$B_{ss,y} = +2\alpha_1\Re(I) - 2\alpha_2\Im(I) \tag{S13}$$

$$B_{ss,z} = -2\alpha_2\Re(I) - 2\alpha_1\Im(I) \tag{S14}$$

$$\alpha_1 = \frac{\mu_0 M}{4\pi}\sin\left(\frac{\pi y}{\lambda}\right)\sinh\left(\frac{\pi z}{\lambda}\right) \tag{S15}$$

$$\alpha_2 = \frac{\mu_0 M}{4\pi}\cos\left(\frac{\pi y}{\lambda}\right)\cosh\left(\frac{\pi z}{\lambda}\right) \tag{S16}$$

where

$$I = \frac{2}{\sqrt{r^2-1}}\left(\arctan\left(\frac{1-r\tan(\frac{\pi w}{4\lambda})}{\sqrt{r^2-1}}\right) - \arctan\left(\frac{1+r\tan(\frac{\pi w}{4\lambda})}{\sqrt{r^2-1}}\right)\right) \tag{S17}$$

and

$$r = \sin\left(\frac{\pi y}{\lambda}\right)\cosh\left(\frac{\pi z}{\lambda}\right) + \mathrm{i}\cos\left(\frac{\pi y}{\lambda}\right)\sinh\left(\frac{\pi z}{\lambda}\right). \tag{S18}$$

S2 Plate approximation

Here, we compare the results for magnetized SpBL plates with different thicknesses t and note that a description in terms of a magnetized sheet is obtained in the limit $t \rightarrow 0$. In the calculations, we keep a fixed value of the plate magnetization M and normalize the calculated average magnetic field by $\tilde{t}$ to account for the variation of the magnetic moment of the plate when t is changed. Figure S2 shows $\langle \tilde{H}_y \rangle / \tilde{t}$ calculated for plates with the indicated values of $\tilde{t}$. For each value of $\tilde{t}$, it is clearly observed that the curves approach a universal 'dipole sheet' curve, which is independent of $\tilde{t}$ and can be obtained for $\tilde{t} \rightarrow 0$, or when $\tilde{z} \geq \tilde{t}/2$ (indicated by the dashed vertical lines in the figure). The inset shows the relative deviation from this universal curve calculated as function of $\tilde{z}_{\mathrm{SpBL}}/\tilde{t}$ for $\tilde{t} = 0.1$. The absolute relative deviation is observed to be below $\sim 10\%$ for $\tilde{z}_{\mathrm{SpBL}} > \tilde{t}/2$ and below 5% for $\tilde{z}_{\mathrm{SpBL}} > \tilde{t}$.

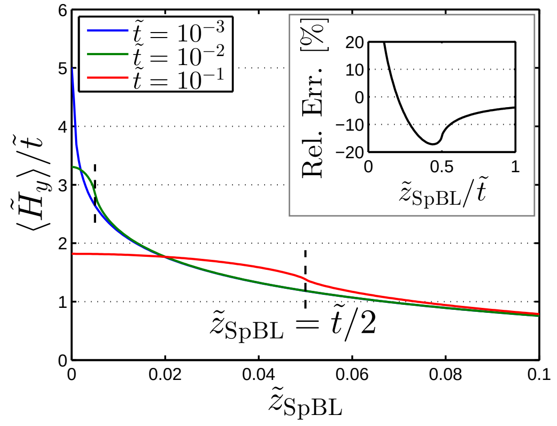

Figure S2. The average field divided by the thickness for the indicated thicknesses of an infinite SpBL array. The dashed lines indicate when the edge of a SpBL passes the sensor. The inset shows the relative error when comparing an infinitely thin sheet to a plate of thickness $\tilde{t} = 0.1$. All calculations were done for $\tilde{w} = 0.5$.

It is clear that the finite thickness of the magnetized plate is only relevant for cases where $\tilde{z}_{\mathrm{SpBL}} < \tilde{t}$. The lowest value of $\tilde{z}$ for beads on top of the sensor area is $\tilde{z} = \tilde{t}/2$ as in this case, the magnetic bead layer is in contact with the sensor layer. Therefore, the effect of a finite bead layer thickness is most relevant for the SpBL. In the limit $\tilde{z}_{\mathrm{SpBL}} < \tilde{t}$, the sensor response is sensitive to the detailed shape of the plate approximation, and the differences between the results obtained for different plate thicknesses arise because the plate corners contribute differently. Thus for $\tilde{z}_{\mathrm{SpBL}} < \tilde{t}$, the plate approximation should be used with some care. It should also be noted that the infinitely thin sheet approximation obtained in the limit $\tilde{t} \rightarrow 0$ should also be used with care as the sheet represents a summation over point dipoles, where each point dipole corresponds to the center of a magnetic bead. In reality, due to the finite bead size, the center of a magnetic bead must be one bead radius away from the sensor. The use of a magnetic sheet extending to the edge of the sensor will therefore overestimate the signal for $\tilde{z}_{\mathrm{SpBL}} \leq \tilde{t}/2$. This is in agreement with the observation in Fig. S2 that the universal 'dipole sheet' curve obtained for $\tilde{t} \rightarrow 0$ diverges for $\tilde{z}_{\mathrm{SpBL}} \rightarrow 0$ and that the sheet results lie above those obtained for a plate of finite thickness. However, the dipoles are smeared out in the plate approximation and thus this approximation is less sensitive to this issue, and we thus find the plate approximation to be better even for $\tilde{z}_{\mathrm{SpBL}} < \tilde{t}/2$. In this work we therefore chose to focus on the plate approximation. We note that a more realistic representation of a bead layer would be in the form of a plate with rounded edges, but this goes beyond the scope of the present work, where we simply note that the quantitative predictions obtained for plates should be used with care when $\tilde{z}_{\mathrm{SpBL}} \leq \tilde{t}/2$.

S3 Parameters of sensor array used by Gaster *et al.*

Figure S3 illustrates the spin-valve sensor array used by Gaster *et al.*[2] Each of the $N = 31$ sensor stripes has a width of $w = 0.75$ µm, a spacing of $s = 0.75$ µm and a length of $L = 100$ µm. The spin valve sensor stripes have a nominal magnetic stack of Ta(5)/Seed layer(4)/MnIr(8)/CoFe(2)/Ru(0.8)/CoFe(2)/ Cu(2.3)/CoFe(1.5)/Ta(3), all thicknesses in nanometers, and is further coated with a protective oxide of SiO_2(10)/Si_3Ni_4(20)/SiO_2(10).[3] This stack has a magnetoresistive ratio of 12 % and the bottom ferromagnetic layer is pinned along the y-direction. For zero external magnetic field, shape anisotropy confines the magnetization of the free top ferromagnetic layer to be along the x-direction. An applied magnetic field rotates the magnetization away from this orientation to provide the signal. Taking the separation from the center of the free top CoFe layer,

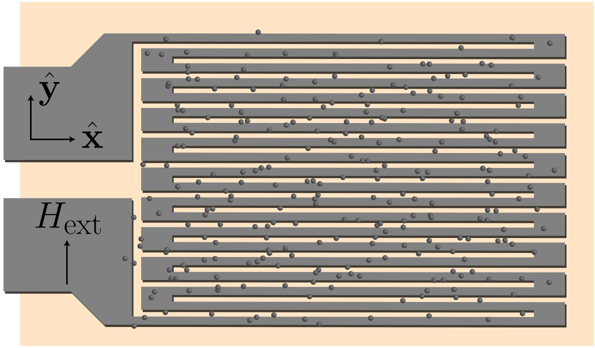

Figure S3. Illustration of the sensor array geometry used by Gaster *et al.*. H_{ext} is the external field for magnetizing the beads. Illustration is not to scale, and the real sensor array had $N = 32$ sensor stripes.

we obtain $h_{\text{SeBL}} = 0.75 + 3 + 40$ nm= 43.75 nm. AFM measurements have revealed a height difference of 60 nm between the top of the sensor stripes and the space between them. This number arises from the stack thickness and the overetching when the stack is structured by use of ion milling. Thus, $h_{\text{SpBL}} = 43.75 - 60$ nm= -16.25 nm. The beads used in experiments are Microbeads from Miltenyi Biotech with a nominal diameter of $t = 50$ nm. These values result in $z_{\text{SeBL}} = h_{\text{SeBL}} + t/2 \approx 69$ nm and $z_{\text{SpBL}} = h_{\text{SpBL}} + t/2 \approx 9$ nm given in Table 1 of the main text.

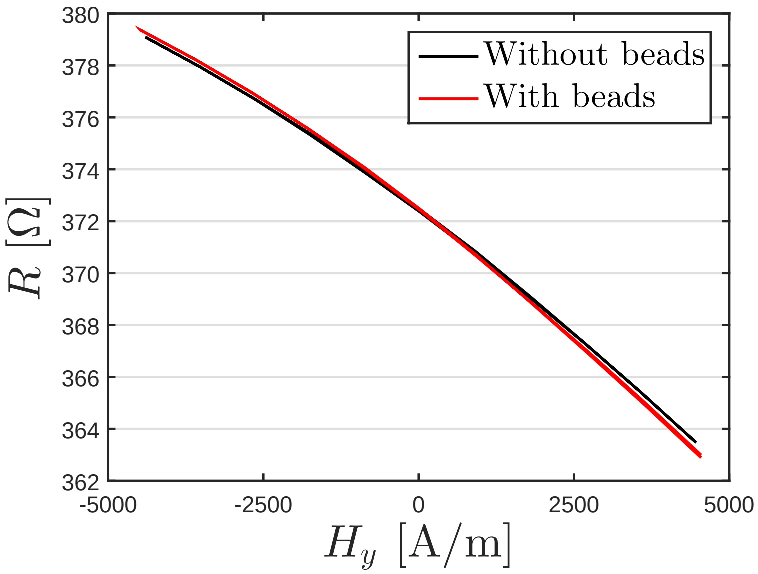

Figure S4. Experimental measurement of the sensor resistance vs. applied magnetic field with and without magnetic beads present. When magnetic beads are present, the slope of the response is larger than when no beads are present. Hence, the effective response from the magnetic beads is along the applied magnetic field, i.e., the beads strengthen the applied magnetic field.

S4 Sign of bead signal

To validate the theory that the SpBL dominates the bead signal, the sensor array used by Gaster *et al.* described in Section S3 was used for an experimental comparison of the magnetic field with and without beads. In the experiments, the sensor chip was exposed to a suspension of Miltenyi Microbeads and a comparison was made between the sensor response vs. field prior to exposure to magnetic beads and after 27 min of exposure to the magnetic bead suspension. Figure S4 shows the sensor resistance, R, measured vs. field for the two cases. It is clearly observed from the measurements that the change in resistance is larger when the sensor is exposed to the magnetic beads. Hence, the magnetic beads *increase* the effective magnetic field experienced by the sensor array. This shows that the signal is dominated by the SpBL in agreement with the theoretical

prediction and hence presents a qualitative validation of the theoretical analysis. We note that if the signal were dominated by the SeBL, the presence of magnetic beads would weaken the external magnetic field and decrease the slope of the curve in Fig. S4 as observed by Li *et al.*[4] for magnetic nanoparticles selectively positioned on top of a sensor stripe with a different dimension than that presented in the previous section. Although a quantitative comparison between the model predictions and the available experimental data is not feasible at present due to lack of knowledge on the amount and distribution of magnetic beads in experiments, we note that the sign of the presented data verify the basic signal mechanism and hence supports the proposed shift of paradigm to maximize the signal due to magnetic beads between sensor stripes.

References

1. Asmar, N. H. *Partial differential equations with Fourier series and boundary value problems* (Pearson Prentice Hall, 2005), 2nd edn.

2. Gaster, R. S. *et al.* Matrix-insensitive protein assays push the limits of biosensors in medicine. *Nat. Med.* **15**, 1327–1332 (2009).

3. Gaster, R. S. *et al.* Quantification of protein interactions and solution transport using high-density GMR sensor arrays. *Nat. Nanotechnol.* **6**, 314–320 (2011).

4. Li, G. *et al.* Spin valve sensors for ultrasensitive detection of superparamagnetic nanoparticles for biological applications. *Sens. Acts. A* **126**, 98 (2006).
